# Case Report: Cannabidiol-Induced Skin Rash: A Case Series and Key Recommendations

**DOI:** 10.3389/fphar.2022.881617

**Published:** 2022-05-19

**Authors:** José Diogo S. Souza, Maíra Fassoni-Ribeiro, Rayssa Miranda Batista, Juliana Mayumi Ushirohira, Antonio W. Zuardi, Francisco S. Guimarães, Alline C. Campos, Flávia de Lima Osório, Daniel Elias, Cacilda S. Souza, AndRea A. Fassoni, Jaime E. C. Hallak, José Alexandre S. Crippa

**Affiliations:** ^1^ Department of Neurosciences and Behavior, Ribeirão Preto Medical School, University of São Paulo, Ribeirão Preto, Brazil; ^2^ Department of Dermatology, São Paulo Federal University, São Paulo, Brazil; ^3^ Division of Dermatology, Department of Internal Medicine, Ribeirão Preto Medical School, University of São Paulo, Ribeirão Preto, Brazil; ^4^ National Institute of Science and Technology—Translational Medicine, Ribeirão Preto, Brazil; ^5^ Department of Pharmacology, Ribeirão Preto Medical School, University of São Paulo, Ribeirão Preto, Brazil; ^6^ Anatomed Laboratory, Bauru, Brazil

**Keywords:** cannabidiol, drug interaction, side effects, skin rash, case report, case series

## Abstract

Cannabidiol (CBD) is a non-psychotomimetic constituent of the *Cannabis* plant, with potential therapeutic properties for many physical and neuropsychiatric conditions. Isolated CBD has been suggested to have favorable safety and tolerability. Although CBD-related rash is described, few case reports are well documented in the literature, and usually, CBD was used concomitantly with other medications. Thus, we report four women who presented a skin rash after ongoing CBD use. Other causes of these skin rashes were ruled out after conducting an extensive viral and serological detection panel, and three patients had their lesions biopsied. Two patients were re-exposed to the vehicle (MCT) without developing a new skin rash. Therefore, clinicians must be aware of this potential adverse effect of CBD use.

## Introduction

Cannabidiol (CBD) is a non-psychotomimetic phytocannabinoid with potential therapeutic properties across many physical and neuropsychiatric conditions (Crippa et al., 2018). CBD is generally well tolerated and has few serious adverse effects (Chesney et al., 2020). There is an increasing requirement in many countries for patients to legally use this cannabinoid to treat sleep disorders, addiction, pain, and anxiety and, more broadly, promote their health and wellbeing (Crippa et al., 2018). However, only the isolated CBD Epidiolex® (GW-Pharm, United Kingdom) has been licensed for two rare childhood epilepsies in some Western countries. Some of the most common side effects of Epidiolex® include sleepiness, decreased appetite, diarrhea, increase in liver enzymes, sleep problems, and cutaneous rash (EPIDIOLEX, 2022). Although this skin condition is documented as a common side event, the trials that reported it had used CBD formulations as an adjuvant treatment to other drugs (Devinsky et al., 2018).

We report here four women, three patients without comorbidities and one with diabetes using daily medications, who presented a skin rash between 6 h and 11 days after starting CBD use. The subjects were volunteers in the Brazilian clinical trial and follow-up study to evaluate the anxiolytic effects of CBD in healthcare frontline workers during the current COVID-19 pandemic, between June and November 2020. Part of these results has been published (Crippa et al., 2021). The subjects received isolated oral CBD (99.6% purity with no other cannabinoid; PurMed Global; United States) dissolved in medium-chain triglyceride oil (150 mg/ml) at a daily dose of 300 mg (150 mg twice per day) for 4 weeks. To the best of our knowledge, these are the first case reports with multiple individuals not using other medications, with an extensive investigation to exclude other clinical causes (Pettit et al., 2018; Singh and Antimisiaris, 2020). Half of the patients were exposed only to the vehicle (medium-chain triglyceride (MCT) oil) to confirm that the adverse effect was specifically related to CBD use.

## Case Description

### Case 1

**FIGURE 1:**
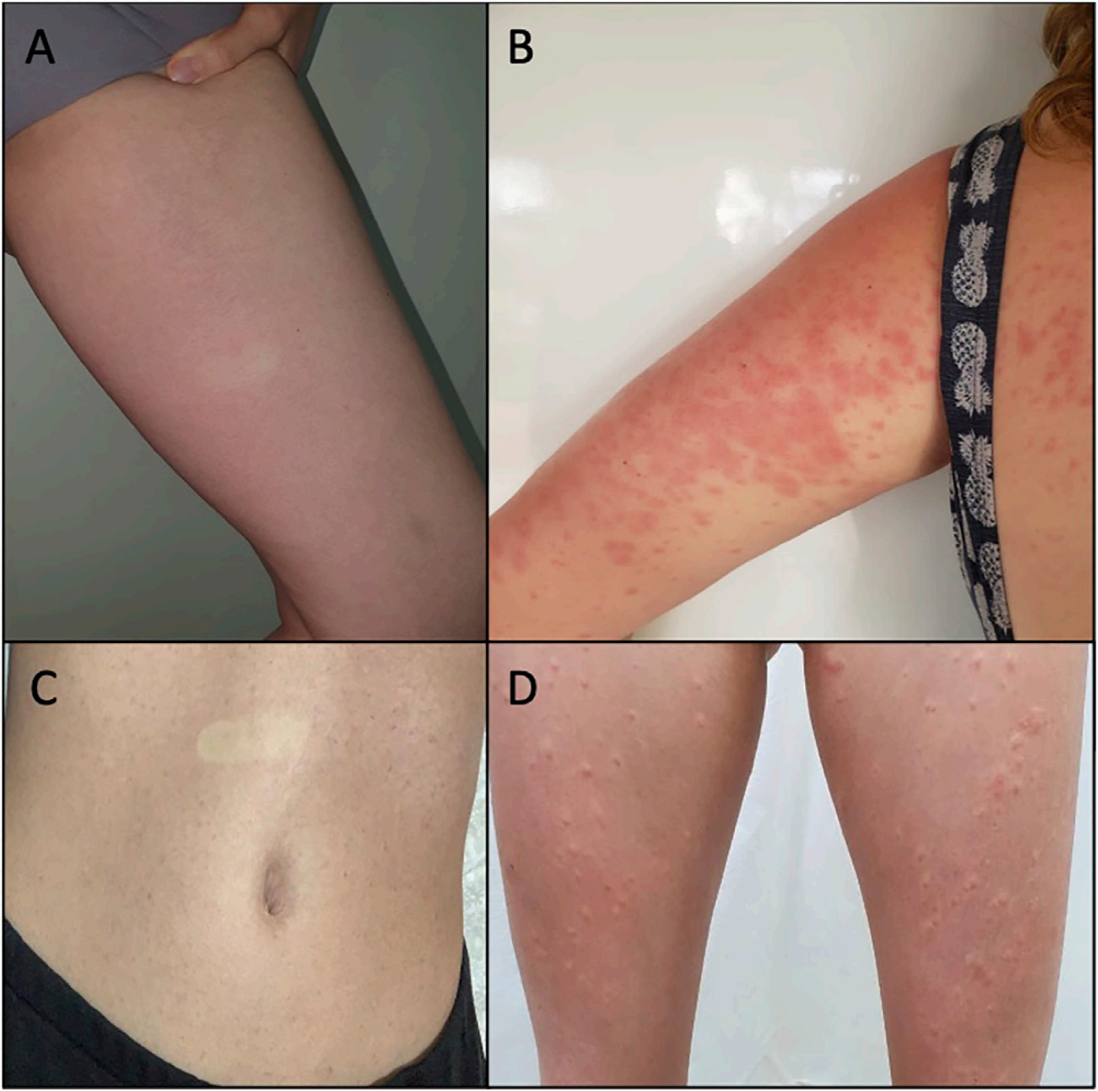
**(A)** Case 1: rash on the inner side of the left thigh after 6 h of CBD use. The disappearance of the lesion on digital pressure is observed. **(B)** Case 2: confluent erythematous papules on the trunk and left arm after 8 days of CBD use. **(C)** Case 3: abdominal rash after 5 days of CBD use. The disappearance of the lesion on digital pressure is observed. **(D)** Case 3: evolution of rash to erythematous papules, surrounded by a hypochromic halo, on the lower limbs, after 10 days of CBD use.

A 34-year-old woman, without comorbidities or medication use, after 6 h of CBD use, noticed the emergence of a skin rash in the cervical region, trunk, and abdomen, with progression to the upper and lower limbs (Figure 1A). She reported an associated feeling of abdominal distension that improved after taking symptomatic medication, Simethicone. She had no fever or any other systemic symptoms. She presented a morbilliform exanthem on the face, neck, trunk, abdomen, upper limbs, and thighs, sparing the face and palmoplantar region, without mucosal lesions. There were no abnormalities in the blood count, and the broad investigation of viral infections and syphilis by RT–PCR or serology was negative for recent or active infections (Table 1). CBD withdrawal resulted in the involution of all lesions after 5 days, without any topical or systemic therapy. The patient voluntarily agreed to be exposed to the CBD vehicle (medium-chain triglyceride (MCT) oil). After 5 days of use, there was no recurrence or emergence of new rashes.

**TABLE 1:** Laboratory findings for investigation of infections in the four patients with skin rash using cannabidiol.

Exams	Case 1	Case 2	Case 3	Case 4
PCR				
Multiplex for dengue, Zika virus, and chikungunya	(−)	(−)	(−)	(−)
Herpes simplex- type I	(−)	(−)	(−)	(−)
Herpes simplex- type II	(−)	(−)	(−)	(−)
Herpes simplex- type IV	(−)	(−)	(−)	(−)
Cytomegalovirus	(−)	(−)	(−)	(−)
Parvovirus	(−)	(−)	(−)	(−)
Enterovirus	(−)	(−)	(−)	(−)
SARS-CoV-2 (PCR)	(−)	(−)	(−)	(−)
Anti-SARS-CoV-2 antibodies	IgM -/IgG -	IgM -/IgG -	IgM -/IgG -	IgM -/IgG -
ELISA
Rubella	IgM -/IgG +	IgM -/IgG +	IgM -/IgG +	IgM -/IgG +
Cytomegalovirus	IgM -/IgG +	IgM -/IgG +	IgM -/IgG +	IgM -/IgG +
Epstein–Barr	IgM -/IgG +	IgM -/IgG +	IgM -/IgG +	IgM +/IgG +
Anti-HIV	(−)	(−)	(−)	(−)
Serologies
Syphilis (VDRL)	(−)	(−)	(−)	(−)
Hepatitis B and C	(−)	(−)	(−)	(−)

### Case 2

A female subject, 33 years old, diabetic using metformin, sibutramine, and semaglutide, 7 days after starting CBD use, reported the appearance of erythematous and pruritic papules on the trunk, abdomen, and thighs, with an increase in the lesions and progression to the arms after 1 day. She had no fever or other systemic symptoms associated with the skin condition. A physical examination showed coalescing erythematous papules on the limbs, trunk, and abdomen (Figure 1B) without affecting other areas such as the face, and the palmoplantar and mucosal regions. There was a complete remission of the lesions after 6 days of CBD suspension, without the need for any topical or systemic therapy. There was no change in the blood count (before and after CBD use), and the broad investigation of viral infections and syphilis by RT–PCR or serology was negative for recent or active infections (Table 1).

### Case 3

A 34-year-old woman without comorbidities or medication use developed a rash on the abdomen after 5 days of CBD use (Figure 1C). The patient chose to continue using CBD, and after 5 days, the skin condition worsened, with progression to erythematous lesions on the buttocks and limbs, which were not pruritic but sensitive. She presented associated headaches and myalgia without fever or other systemic complaints. On physical examination, monomorphic erythematous papular lesions with a hypochromic halo were observed on the trunk, back, buttocks, lower limbs, and upper limbs (Figure 1D), sparing the face and the palmoplantar region, and without mucosal lesions. Withdrawal of CBD resulted in the involution of all lesions after 7 days, without the need for any topical or systemic therapy. There was no change in the blood count (before and after CBD use), and the broad investigation of viral infections and syphilis by RT-PCR or serology was negative for recent or active infections (Table 1). The patient voluntarily agreed to be exposed to the CBD vehicle (MCT), and after 5 days of use, there was no recurrence or emergence of new rashes.

### Case 4

**FIGURE 2:**
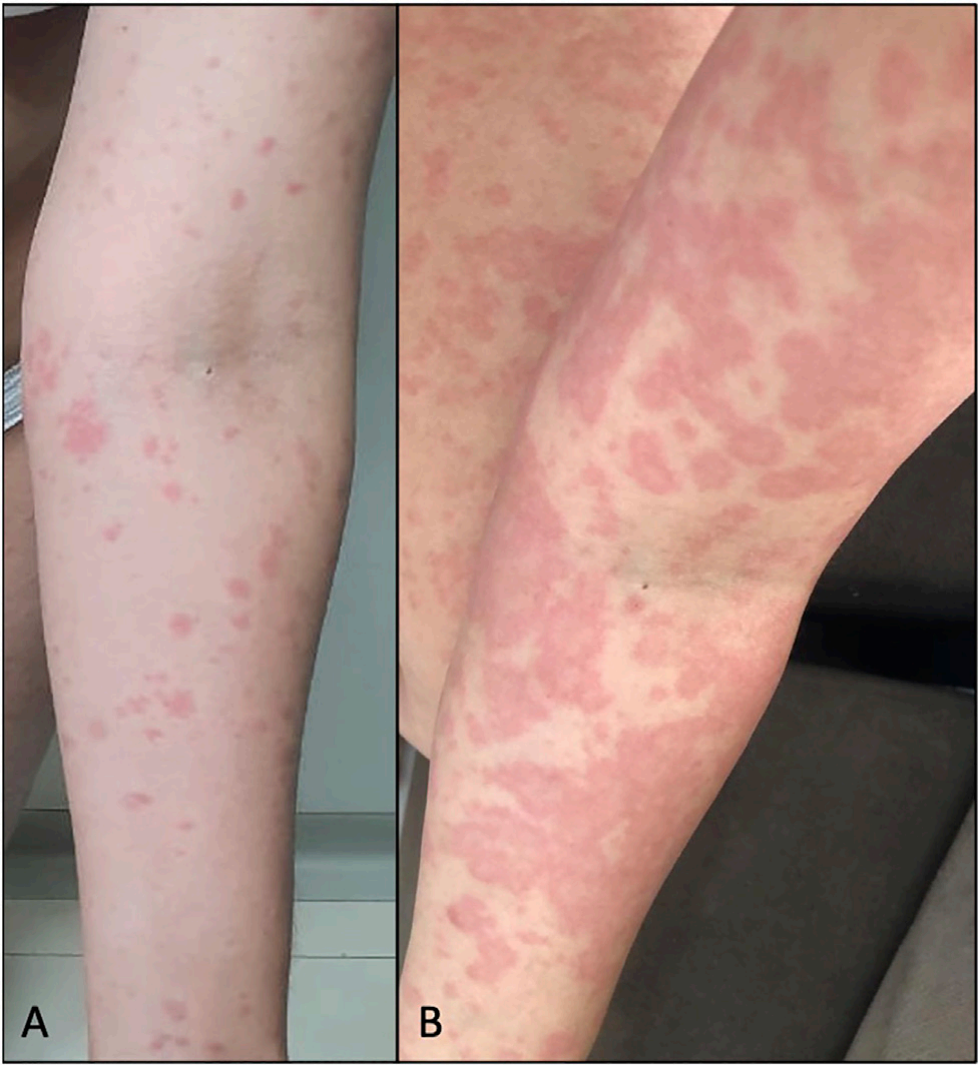
**(A)** Case 4: erythematoedematous papules on the left upper limb after 9 days of CBD use. **(B)** Evolution of the lesions after 2 days, after medication discontinuation, and initiation of oral prednisone.

A female subject, 28 years old, without comorbidities or medication use, reported erythematous and pruritic lesions on the trunk and abdomen with evolution to arms and legs after 9 days of CBD use. She had associated diarrhea but did not present fever or other systemic symptoms. The physical examination showed monomorphic urticarial lesions (Figure 2A) that evolved after 2 days, with coalescence in the trunk, abdomen, and the upper and lower limbs (Figure 2B), even after discontinuing CBD in the period. Lesions spared the face and the palmoplantar and mucosal regions. There was a complete improvement of the lesions after 11 days of discontinuation of CBD, and it was necessary to initiate oral corticosteroid therapy (prednisone 0.5 mg/kg/day). There was no change in the blood count (before and after CBD use). The broad investigation of viral infections and syphilis by RT–PCR or serology was negative for other active or recent infections (Table 1), except for IgM positivity for Epstein–Barr.

## Discussion

Adverse drug reactions focus on clinical studies to connect possible side effects and the studied compound. Skin lesions often herald these reactions because this body organ can be quickly evaluated and confirmed to be immunologically active. These aspects make it particularly relevant in this and other clinical contexts. However, the skin condition itself can be highly non-specific, and it is always necessary to rule out other possible clinical causes, especially viral infections and drug vehicle allergies.

These skin reactions can be considered pharmacologically mediated (type A) or allergic or non-allergic hypersensitivity reactions (type B) (Böhm et al., 2018). Type A reactions, as they are generally dose dependent, can be resolved with changes in therapy and allow for the maintenance of treatment. On the other hand, Type B reactions constitute the minority, although they are more unpredictable than type A. Moreover, these responses can lead to severe conditions, such as Stevens–Johnson syndrome (SJS), toxic epidermal necrolysis (TEN), or drug reaction with eosinophilia and systemic symptoms (DRESS). In these cases, re-exposure to the potentially causative drug is strictly not recommended.

The most common form of drug rash is morbilliform or maculopapular (Hoetzenecker et al., 2016). Nonetheless, the same drug can cause different patterns of dermatological manifestation since the pathogenesis of pharmacodermia is multifactorial and may involve genetic factors, lymphocyte activation, metabolic enzyme defects, eosinophilia, herpes virus activation, among others. Regarding DRESS cases, it is believed that an allergic immune response to a particular drug can stimulate T cells and induce viral reactivation (Husain et al., 2013). In one of the present study cases, even though it was a mild skin reaction, positive IgM and IgG for Epstein–Barr may suggest a relationship between pharmacodermia and reactivation of herpes virus 7 (HHV-7).

We report four women, three patients without comorbidities, and one with diabetes using daily medications, who presented with skin rashes between 6 h and 9 days after starting CBD use. The experimental CBD studied was pharmaceutical grade with no other compound than the MCT oil vehicle. In total, 100 participants (79 women and 21 men) received CBD oil, with four skin reactions related to the medication use (4% of volunteers). Three main patterns were observed: exanthematous, monomorphic erythematous papular lesions, and urticarial pattern, on the trunk and abdomen, with centrifugal progression to the limbs, sparing the face and palmoplantar regions, which were pruritic or sensitive to touch. There was an association with headache and myalgia in one case and abdominal symptoms in two other cases. The suspension of CBD resulted in the involution of the lesions between 5 and 11 days, without any topical or systemic therapy for three of the patients and prednisone 0.5 mg/kg for 5 days for one of the patients. Subsequent re-exposure to CBD vehicle (MCT) for 5 days in two of the patients did not induce a recurrence of the rash.

**FIGURE 3:**
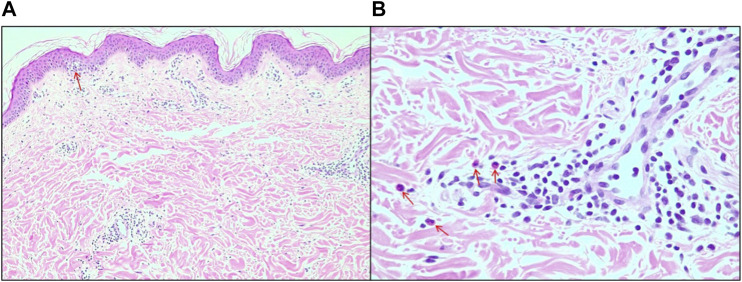
**(A)** Edema of the papillary dermis and focal area of vacuolar degeneration of the basal layer of the epidermis (arrow), 10x magnification. **(B)** Details for the presence of eosinophils (arrows), 20x magnification.

A broad investigation of viral infections and serology for syphilis and hepatitis was carried out, which were negative, except for one of the patients who had positive serology for the Epstein–Barr virus. The relevance of research on arboviruses is worth noting since they are prevalent in the study region and on SARS-CoV-2, given the context of the COVID-19 pandemic. In addition, lesions were biopsied from three patients (cases 1, 3, and 4). The histopathological pattern showed skin with mild hyperkeratosis, dermal edema, focal vacuolar degeneration of the basal layer, and lymphohistiocytic infiltrate with sparse eosinophils predominantly in the perivascular and periadnexal locations (Figure 3). Given such clinical, laboratory, and anatomopathological findings, the four cases can be diagnosed as skin rashes induced by CBD use. To the best of our knowledge, these are the first case reports with multiple individuals not using other medications, with an extensive investigation to exclude other clinical causes and half of the patients being exposed only to the MCT vehicle afterward (Pettit et al., 2018; Singh and Antimisiaris, 2020).

An individual analysis of CBD plasma levels was only possible in two of the subjects since the other two withdrew from the trial before measurement. These two subjects showed levels that reached the upper 95% percentile of the whole study (levels of 98 and 179 ng/ml, respectively). This finding suggests that the reported pharmacodermia could be associated with high plasma levels of CBD. However, this possibility should be seen with caution, considering the low number of subjects. Also, the cutaneous rash did not occur in five other subjects that also reached similar high CBD plasma levels. CBD is extensively metabolized by CYP450 isoenzymes. The extent to which genetic polymorphisms in these isoenzymes would affect CBD plasma levels and their side effects remains poorly explored and should be a focus of future research.

Cannabis products are widely employed worldwide, and their use has been illegalized in several countries (Bonomo et al., 2018). After legalizing medicinal and recreational cannabis in some countries, its use highly increased (Wen et al., 2015; Martins et al., 2016; Hasin et al., 2017). In the United Kingdom, for instance, it is estimated that over a million people are using non-legally sourced cannabis for self-medication of diagnosed conditions (Schlag et al., 2021). CBD has gained special attention for its therapeutic potency for several neuropsychiatric and general medical conditions, including a trend as a new ingredient for skincare products. However, apart from epilepsy, much of the available data are from preclinical studies and lack the support of robust human, randomized, placebo-controlled, double-blind studies (Jhawar et al., 2019; Baswan et al., 2020).

While there is a growing demand in many countries for legal CBD use to treat various mental and physical health problems, determining the best regulatory response to these demands is a significant challenge. It occurs because most CBD products come in multiple formulations and routes of administration, and the great majority is prepared in an artisanal manner, without the necessary manufacturing, quality control, labeling, and pharmaceutical-grade production. In addition, there is a paucity of data to support the therapeutic use of many of the indications consumed by this tremendous global demand. The clinical use of CBD needs to be aligned with many other pharmaceutical products to ensure efficacy and safety for patients, given its interactions with other drugs and its potential side effect profile. Therefore, through evidence-based medicine, an exhaustive framework to guarantee the quality and safety of the CBD product is urgently needed to protect the consumers and minimize possible harm.

## Conclusion

We report four cases who presented with a skin rash between 6 h and 11 days after starting CBD use. Considering the clinical, laboratory, and anatomopathological findings, the four cases can be diagnosed as skin rashes induced by this cannabinoid. This finding reinforces the proposal that CBD use should be guided by evidence-based medicine, being necessary to balance the benefits with potential adverse and undesired effects when making decisions regarding its use.

## Data Availability

The raw data supporting the conclusions of this article will be made available by the authors if requested, without undue reservation.
